# Unplugging the Drain: Treatment of a Rare Late Complication of Cardiac Resynchronization Therapy

**DOI:** 10.7759/cureus.4527

**Published:** 2019-04-23

**Authors:** Faheemullah Beg, Tanushree Agrawal, Ashrith Guha

**Affiliations:** 1 Cardiovascular Disease, Houston Methodist Hospital, Houston, USA; 2 Internal Medicine, Houston Methodist Hospital, Houston, USA; 3 Heart Failure and Transplant Cardiology, DeBakey Cardiology Associates, Houston Methodist Hospital, Houston, USA

**Keywords:** subclavian vein, cardiac resynchronization therapy, anticoagulation, thrombosis

## Abstract

Central vein thrombosis (CVT) is a rare cause of chylothorax. We report a patient with chylothorax secondary to CVT, despite being on anticoagulation, who was successfully treated with balloon angioplasty of the occluded vein. A 65-year-old male presented with shortness of breath of one-month duration. He had end-stage heart failure and was on milrinone infusion. Physical exam was consistent with elevated central venous pressure. Chest x-ray showed a large left-sided pleural effusion. Right heart catheterization (RHC) showed normal right atrial pressure and normal pulmonary capillary wedge pressure. Diagnostic thoracentesis was consistent with chylothorax. A venogram revealed bilateral brachiocephalic vein occlusion. The right brachiocephalic vein was recanalized by angioplasty, which led to resolution of pleural effusion. Our case not only highlights the identification of a rare complication of a common device (i.e., cardiac resynchronization therapy defibrillator) used in heart failure patients but also highlights the treatment for this rare but reversible complication.

## Introduction

Subclavian vein occlusion occurs in up to 30% of patients after cardiac resynchronization therapy defibrillator (CRT-D) placement [1]. This occlusion, however, tends to be asymptomatic in most cases because of collateral vessel development. Central vein thrombosis (CVT) and occlusion is a rare cause of chylothorax. Its exact incidence is unknown, but a few case reports exist [2-5]. We report a unique case of chylothorax and bilateral CVT related to CRT-D on the left and a peripherally inserted central venous line on right side. The patient was successfully treated with balloon angioplasty of the right brachiocephalic vein.

## Case presentation

**Figure 1:**
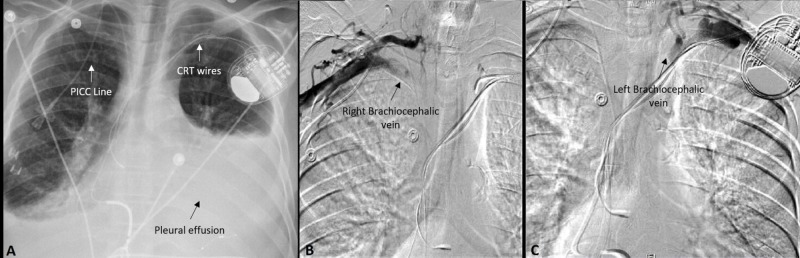
Imaging prior to intervention Large left-sided pleural effusion seen on chest x-ray (A) prior to angioplasty. Right-sided PICC (peripherally inserted central venous line) and left-sided CRT (Cardiac resynchronization therapy) wires can be appreciated as well. Occlusion of the right (B) and left (C) brachiocephalic veins can be seen on the venogram.

A 65-year-old male with end-stage heart failure presented with worsening shortness of breath for the past month. He had a history of atrial fibrillation and ischemic cardiomyopathy and was currently on milrinone infusion and post-CRT-D placement. At the time of admission, his vital signs were within normal limits. The physical exam was most notable for an elevated jugular venous pressure (JVP) up to the angle of the jaw and decreased breath sounds on the left side of the chest. A chest x-ray showed a large left-sided pleural effusion as shown in Figure 1A. Initial escalation of diuretic therapy led to acute kidney injury. During right heart catheterization (RHC), the operator reported apparent right internal jugular vein (IJV) thrombosis based on an ultrasound exam. After cannulation of the left IJV and upon contrast injection, complete occlusion of the left subclavian vein was noted. RHC via femoral access revealed normal right heart filling pressure (right atrial pressure 6 mmHg), normal pulmonary capillary wedge pressure (10 mmHg), and decreased cardiac index (1.94 L/min/m2). Next, ultrasound evaluation of the right subclavian vein showed partial thrombosis. The patient was already on therapeutic anticoagulation for atrial fibrillation, so this treatment was continued to treat the subclavian deep vein thrombosis.

The left-sided pleural effusion noted at admission was evaluated via diagnostic thoracentesis; this yielded 2 L of cloudy fluid, so a pigtail catheter for continuous drainage was placed. The pleural fluid studies were normal with the exception of triglycerides (1177 mg/dL), which was consistent with chylothorax. Because of continued high output (1-2 L/day) from the chest tube, the patient was started on a low-fat diet.

**Figure 2:**
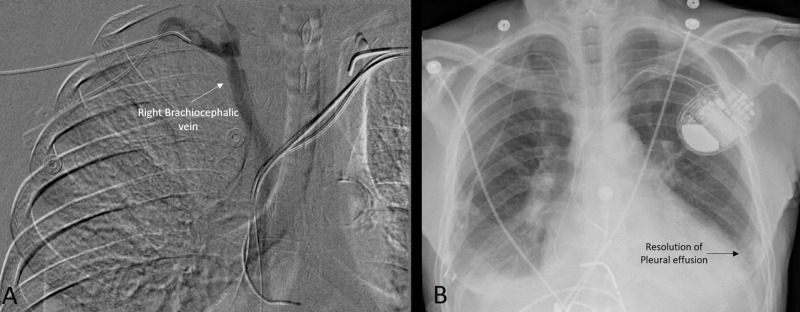
Imaging after the intervention The flow of contrast through the right brachiocephalic vein and superior vena cava can be seen (A) post balloon angioplasty. This led to near complete resolution of the left-sided pleural effusion as seen on chest x-ray (B).

Next, a venogram was used to assess for venous obstruction as a possible cause of the chylothorax. The venogram revealed occlusion of bilateral brachiocephalic veins as shown in Figures 1B-1C. The occluded right brachiocephalic vein was successfully recanalized after angioplasty using a 12 mm balloon as shown in Figure 2A. However, CRT-D wires prevented intervention on the left brachiocephalic vein. After this intervention, chest tube output decreased to a negligible amount, so the tube was removed.

For the next five days, the patient was monitored with serial chest x-rays, but there was no reaccumulation of pleural effusion as shown in Figure 2B. Therefore, we decided not to intervene upon the left brachiocephalic vein thrombosis. The patient was eventually discharged home on apixaban. 

## Discussion

Chylothorax is the accumulation of chyle in the pleural space. It is diagnosed based on the presence of pleural fluid triglyceride concentration greater than 110 mg/dL or the detection of chylomicrons on pleural fluid electrophoresis [6]. The etiology of chylothorax can be broadly divided into two groups: traumatic and non-traumatic [7-9]. Traumatic chylothorax is usually associated with surgical procedures or penetrating trauma. Non-traumatic chylothorax has a broad differential diagnosis. The most common causes of non-traumatic chylothorax are malignancy and infiltrative diseases [7-9]. Central venous occlusion is an exceedingly rare cause of chylothorax in adults [2-5]. Elevated central venous pressures likely result in elevated lymphatic pressures in the thoracic duct leading to the rupture of lymphatic vessels. Unilateral vein thrombosis is usually silent due to collaterals, but the presence of bilateral disease is more likely to result in such a drastic presentation. This idea is supported by the observation that our patient’s symptoms resolved after a unilateral intervention.

Management strategies are broadly categorized into conservative and surgical treatment [8-10]. Conservative treatment entails replacing nutrients lost in chyle, maintaining a low-fat diet, and supplementing with medium-chain triglycerides. Surgical modalities to treat chylothorax include thoracic duct ligation, percutaneous thoracic duct embolization, pleurodesis, and creation of a pleuroperitoneal shunt.

## Conclusions

Amongst the reported cases of chylothorax resulting from central venous occlusion in adults, anticoagulation was the primary mode of treatment. To the best of our knowledge, this is the first case report wherein angioplasty of the occluded central vein resulted in complete resolution of chylothorax in an adult.
